# Correlation between the reduction of IL-6 and TNF-α with muscle mass restoration in hyperthyroid patients post-radioactive iodine therapy

**DOI:** 10.3389/fendo.2026.1794009

**Published:** 2026-08-21

**Authors:** Yuqian Ren, Cuijuan Zheng, Meng Wang, Yanzhi Wang

**Affiliations:** 1 School of Basic Medicine, Qingdao University, Qingdao, China; 2 Department of Traditional Chinese Medicine, Qingdao United Family Hospital, Qingdao, China; 3 Department of Pharmacy, Linyi People’s Hospital, Linyi, China; 4 Office of Academic Affairs, Binzhou Medical University, Yantai, China

**Keywords:** hyperthyroid, IL-6, muscle, sarcopenia, TNF-α

## Abstract

**Objective:**

To investigate whether dynamic resolution of IL-6 and TNF-α after radioactive iodine (RAI) therapy is a key determinant of muscle recovery in Graves’ disease and to evaluate their potential as longitudinal biomarkers.

**Methods:**

A prospective cohort of 157 patients with Graves’ disease was enrolled. Skeletal muscle index (SMI) was assessed by dual-energy X-ray absorptiometry (DXA), and serum IL-6 and TNF-α were measured by enzyme-linked immunosorbent assay (ELISA). Patients were re-evaluated 6 months after 131 I therapy. Gaussian graphical modeling (GGM) assessed interaction network centrality, linear mixed models (LMM) evaluated longitudinal effects, and random forest modeling quantified the predictive importance of Δ values for ΔSMI.

**Results:**

At baseline, IL-6 and TNF-α levels were higher in participants with adverse muscle-status phenotypes than in those with normal muscle mass (*P* < 0.05). Both markers were negatively correlated with SMI (IL-6: *r*=−0.19; TNF-α: *r*=−0.23; both *P* < 0.05) and showed high strength centrality in the exploratory network analysis. Among the 29 participants with low muscle mass at baseline, 7 (24.1%) no longer met the low-muscle-mass criterion at 6 months. However, neither within-participant nor between-participant levels of IL-6 or TNF-α were significantly associated with SMI in the mixed-effects models. Changes in IL-6 and TNF-α were also not associated with ΔSMI in correlation or adjusted regression analyses, and natural spline models provided no evidence of nonlinear or threshold relationships. The exploratory random forest ranked ΔFT3 and ΔFT4 highest in permutation importance, but its cross-validated performance was poor (mean R²=−0.204), indicating no reliable out-of-sample predictive ability.

**Conclusion:**

IL-6 and TNF-α were associated with poorer skeletal muscle status at baseline in patients with Graves’ hyperthyroidism, but their longitudinal changes did not independently predict SMI recovery after RAI therapy. Although skeletal muscle mass improved in some patients, neither inflammatory nor thyroid-function changes demonstrated clinically useful predictive performance in this cohort. Larger studies with repeated assessments and external validation are needed to clarify the temporal and mechanistic relationships among thyroid recovery, inflammation, and skeletal muscle restoration.

## 1 Background

Hyperthyroidism, commonly referred to as thyrotoxicosis, is a clinical syndrome caused by excessive secretion of thyroid hormones due to various etiologies. It can occur at any age but is most prevalent in women aged 20–40 years. The primary causes include immune dysfunction, genetic predisposition, and stress factors (1). Elevated basal metabolic rate leads to characteristic symptoms such as heat intolerance, hyperhidrosis, warm and moist skin, fatigue, low-grade fever, nervousness, irritability, emotional excitability, and hand tremor (1). Hyperthyroidism is a catabolic disorder that may cause clinically meaningful loss of skeletal muscle mass and function. Weight loss is a characteristic manifestation of hyperthyroidism, and previous studies suggest that early weight loss may predominantly reflect depletion of lean and skeletal muscle mass rather than loss of fat mass (2). Compared to secondary sarcopenia associated with type 2 diabetes, hyperthyroidism-related sarcopenia has received less research attention. Brennan et al. (3) demonstrated that both overt and subclinical hyperthyroid patients exhibited significantly reduced knee extensor and flexor strength as well as lower thigh muscle cross-sectional area before treatment. Following antithyroid therapy, muscle strength and cross-sectional area improved markedly in overt hyperthyroidism and partially in subclinical cases. Similarly, Riis et al. (4) reported 10–20% reductions in muscle mass and function in hyperthyroid patients prior to treatment, with complete normalization after euthyroidism was achieved. In a veterinary study of 462 untreated hyperthyroid cats, Peterson et al. (2) observed widespread muscle wasting accompanying weight loss; post-treatment reassessment of 117 cats revealed that while most regained weight, approximately half failed to restore normal muscle mass, suggesting independent contributions of hyperthyroidism and aging to persistent muscle deficit. Using the 2014 Asian Working Group for Sarcopenia criteria, clinical studies have reported sarcopenia prevalence is highly in hyperthyroid patients, notably, affected individuals are predominantly in the 40–50-year age range, with skeletal muscle decline observed even in patients over 20 years old, suggesting that age thresholds typically applied in primary sarcopenia may not be appropriate for hyperthyroidism-related cases (5).

Early observations recognized muscle involvement in hyperthyroidism, but mechanistic understanding remained limited until recently (6, 7). Emerging evidence positions skeletal muscle as a key target of thyroid hormone action. Thyrotoxicosis disrupts triiodothyronine (T3), thyroxine (T4), free triiodothyronine (FT3), free thyroxine (FT4), and thyroid-stimulating hormone (TSH) levels, accelerating whole-body protein turnover and promoting muscle catabolism (1, 2). Animal models provide supportive insights: Yamano et al. (8) showed that T4 induces premature muscle remodeling during flounder metamorphosis, an effect inhibited by thiourea. Joseph et al. (9) highlighted that endocrine dysregulation—including reduced sex hormones and insulin-like growth factor-1 (IGF-1), alongside elevated peripheral cytokines and inflammatory/coagulation markers—increases sarcopenia and frailty risk. Hyperthyroidism enhances muscle amino acid release and protein breakdown (4), alters fiber-type distribution (e.g., type I hypertrophy and type II atrophy in hypothyroidism, with opposite patterns suggested in hyperthyroidism) (10), and modulates myosin heavy chain (MHC) expression. Excess T4 impairs contractility, while T3 differentially regulates MHC isoforms (upregulating MHC1, MHC2, MHC4; downregulating MHC7), potentially reducing contractile protein levels and contributing to sarcopenia (11–13). T3 serves as a critical regulator of skeletal muscle metabolism and homeostasis. Hyperthyroidism increases metabolic rate, oxygen consumption, and heat production, whereas hypothyroidism reduces it. T3 significantly alters expression of multiple target genes in skeletal muscle without upregulating type 2 iodothyronine deiodinase (Dio2) (14). T3 administration prevents starvation-induced atrophy independently of forkhead box O3 (FOXO3), proteasome, or autophagy pathways and without marked regulation of atrogin-1 or muscle RING-finger protein-1 (MuRF1), suggesting alternative mechanisms such as phosphatidylinositol 3-kinase/Akt (PI3K/Akt) signaling (14). Additionally, T3 inhibits autophagy to counteract muscle wasting. However, excessively high T3 levels suppress satellite cell proliferation, promote premature differentiation, and impair regeneration, while thyroid hormone receptor α protects satellite cells and supports repair (14, 15). Thus, both hyper- and hypothyroid states disrupt T3 homeostasis and contribute to muscle loss, reduced quality, and diminished strength.

Hyperthyroid patients exhibit markedly elevated serum levels of pro-inflammatory cytokines, including interleukin-6 (IL-6) and tumor necrosis factor-α (TNF-α) (16). In Graves’ disease, IL-6 originates primarily from intrathyroidal lymphocytes rather than follicular cells, with heightened expression in hyperthyroid follicles (17). Pro-inflammatory IL-6 and TNF-α are pivotal inflammatory drivers in the pathogenesis of sarcopenia, promoting skeletal muscle protein catabolism, suppressing protein synthesis, and inducing muscle fiber loss through multiple mechanisms. IL-6 directly contributes to skeletal muscle proteolysis, antagonizes the anabolic and anti-inflammatory actions of insulin-like growth factor-1 (IGF-1), and induces insulin resistance, thereby impairing muscle protein synthesis and calcium uptake, ultimately leading to reductions in muscle mass and strength (18–23). In parallel, TNF-α inhibits the Akt/mTOR signaling pathway to suppress protein synthesis, attenuates the protective effects of IGF-1 on skeletal muscle, activates TRAF6-mediated expression of atrogenes (Atrogin-1/MAFbx and MuRF1) to accelerate myosin heavy chain degradation, and promotes myocyte apoptosis while inhibiting myoblast fusion, thereby exacerbating the progression of sarcopenia (24–29). These cytokines synergistically amplify inflammatory cascades, playing critical pathogenic roles in both primary (age-related) and secondary forms of sarcopenia.

In summary, hyperthyroidism-associated sarcopenia arises from complex interplay of thyroid hormone excess, chronic inflammation, oxidative stress, and potential sex hormone dysregulation. Compared to extensively studied diabetes-related sarcopenia, this entity remains under-investigated. The clinical spectrum of Graves’ hyperthyroidism extends beyond hormonal dysregulation, frequently manifesting as a rapid depletion of skeletal muscle mass (1, 30). While the role of pro-inflammatory cytokines (e.g., IL-6 and TNF-α) in driving muscle proteolysis is well-established in aging, their specific impact on hyperthyroidism-induced sarcopenia remains under-investigated, especially in non-geriatric cohorts (2, 9). The prospective longitudinal design of this study allowed us to examine both baseline associations and within-participant changes after RAI treatment. IL-6 and TNF-α were selected *a priori* because they are among the most extensively studied pro-inflammatory cytokines in Graves’ hyperthyroidism and skeletal muscle catabolism, and validated assays were available when the study was initiated. The study was not designed as comprehensive inflammatory profiling. We prospectively evaluated whether changes in IL-6 and TNF-α were associated with changes in skeletal muscle mass after RAI treatment, without prespecifying these cytokines as causal determinants or validated predictors of muscle recovery.

## 2 Objects and methods

### 2.1 Study population (objects)

**Figure 1:**
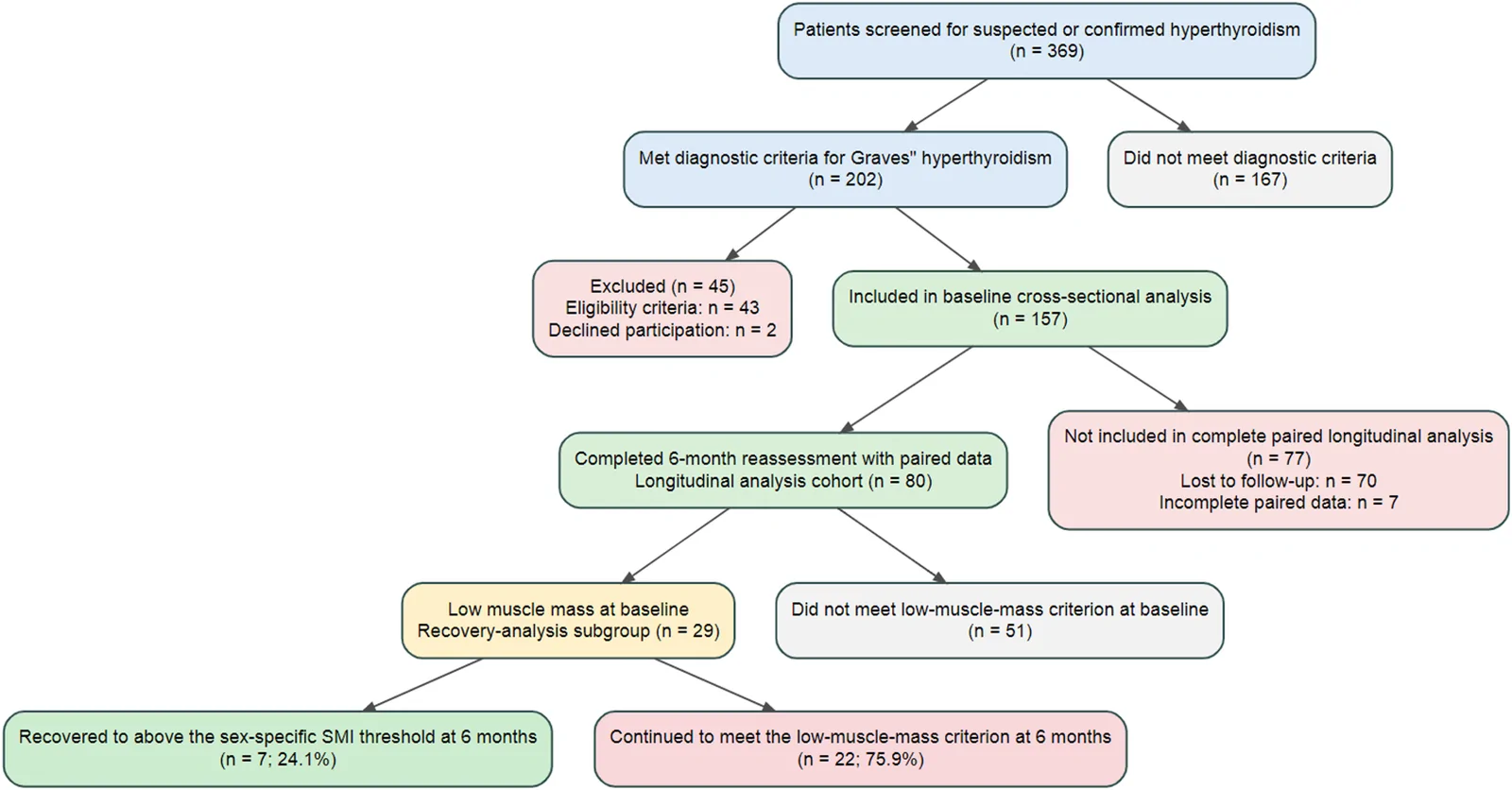
Participant screening flowchart.

Between January 2017 and December 2018, 369 consecutive patients with suspected or confirmed hyperthyroidism were screened at Linyi People’s Hospital. Of these, 202 met the diagnostic criteria for Graves’ hyperthyroidism. Graves’ hyperthyroidism was diagnosed by suppressed serum TSH accompanied by elevated FT3 and/or FT4, together with at least one of the following: positive TSH-receptor antibodies, diffusely increased uptake on thyroid scintigraphy, or diffuse goiter with increased vascularity on ultrasonography. After 43 patients were excluded according to the eligibility criteria and 2 declined participation, 157 participants were included in the baseline cross-sectional analysis. Of the 157 participants, 80 completed the 6-month reassessment and had paired measurements available for the longitudinal analysis. Seventy participants were lost to follow-up, and seven attended follow-up but had incomplete paired data for the primary longitudinal variables. Among the 80 participants in the longitudinal cohort, 29 had low muscle mass at baseline and were included in the low-muscle-mass recovery analysis. Recovery was defined as an SMI at 6 months that no longer met the sex-specific low-muscle-mass threshold. Participant selection is summarized in **Figure 1**. To assess potential attrition bias, baseline demographic, thyroid-related, inflammatory, and muscle-related characteristics were compared between participants who completed the 6-month reassessment and those who did not.

Exclusion criteria:(1) Presence of active malignancy or other severe malignant diseases; (2) Comorbid major chronic diseases affecting skeletal muscle mass or function, including but not limited to: type 2 diabetes mellitus, liver cirrhosis, chronic kidney disease (eGFR <30 mL/min/1.73m²), rheumatoid arthritis, severe chronic obstructive pulmonary disease, congestive heart failure, active tuberculosis, HIV infection, or chronic inflammatory bowel disease; (3) Comorbid major neurological disorders that may directly cause muscle atrophy or impair strength/gait assessment, including but not limited to: post-stroke sequelae, Parkinson’s disease, motor neuron disease, multiple sclerosis; (4)Endocrine disorders other than Graves’ hyperthyroidism that could affect muscle mass or protein metabolism, including Cushing’s syndrome, primary aldosteronism, acromegaly, uncontrolled diabetes mellitus, or other clinically significant pituitary, adrenal, or parathyroid disorders; patients with non-Graves causes of thyrotoxicosis or pre-existing hypothyroidism were also excluded; (5)High risk of disuse atrophy, including: recent (within 6 months) surgical fractures leading to prolonged bed rest, major trauma, extensive burns, prolonged ICU stay, or recovery period after major surgery; (6)Long-term use (>3 months) of medications known to significantly affect skeletal muscle, including but not limited to: glucocorticoids, statins, certain antiepileptic drugs, or ongoing chemotherapy; (7)Presence of moderate-to-severe cognitive impairment or severe psychiatric disorders (e.g., moderate-to-severe dementia, severe depression) that prevent cooperation with grip strength, gait speed, muscle mass measurements, or questionnaire assessments; (8)Any other conditions deemed by the investigator to significantly interfere with sarcopenia diagnosis or intervention effects.

Appendicular skeletal mass was measured by DXA, and the appendicular skeletal muscle index was calculated as ASM divided by height squared (kg/m²). According to the 2014 Asian Working Group for Sarcopenia (AWGS) (31) thresholds, low muscle mass was defined as SMI <7.0 kg/m² in men or <5.4 kg/m² in women, and low handgrip strength was defined as <26 kg in men or <18 kg in women. Gait speed was not measured; therefore, physical performance could not be comprehensively assessed. Participants were categorized operationally as having: (1) normal muscle mass; (2) low muscle mass with preserved handgrip strength; or (3) low muscle mass combined with low handgrip strength. Because the AWGS criteria were developed primarily for older Asian populations and the present cohort included adults aged 18–80 years, these categories were interpreted as operational phenotypes of Graves’ hyperthyroidism-associated muscle loss rather than definitive evidence of primary age-related sarcopenia. Handgrip strength was measured with participants in a standardized seated position using the dominant hand. A total of two measurements were obtained with at least 30 seconds of rest between attempts, and the maximum value was used in the analysis. The dynamometer was calibrated according to the manufacturer’s instructions.

The study protocol was approved by the Medical Ethical Review Board of the Linyi People’s Hospital, and written informed consent was obtained from all participants.

### 2.2 Clinical and biochemical measurements

Body Composition: Skeletal muscle mass and fat-free mass were measured via Dual-energy X-ray Absorptiometry (DXA; GE, USA). The Skeletal Muscle Index (SMI) was calculated as Appendicular Skeletal Mass(ASM)/height^2^(m^2^).

Muscle Function: Handgrip strength was assessed using a calibrated dynamometer (EH101, Xiangshan, China).

Biomarkers: Serum IL-6 and TNF-α were quantified via Enzyme-Linked Immunosorbent Assay (ELISA; Kits SUB10377 & SUB11776, Shanghai, China). The intra-assay and inter-assay coefficients of variation (CV) were <10% and <15%, respectively.

Follow-up: Patients were reassessed 6 months post-radioactive iodine 131 (RAI) treatment to evaluate muscle recovery and biomarker shifts.

### 2.3 Radioactive iodine treatment

All participants received a single oral administration of iodine-131. The administered activity was individualized according to the institutional treatment protocol. The calculation considered body weight, estimated thyroid mass or volume, 24-hour radioactive iodine uptake, the severity and duration of hyperthyroidism, previous antithyroid treatment, and relevant clinical characteristics. The prescribed activity was calculated using the following institutional formula: Administered activity= target activity per gram×estimated thyroid mass/24-hour uptake fraction.

### 2.4 Statistical analysis

Data were expressed as mean ± SD, median (IQR), or frequencies as appropriate. Initial comparisons utilized Student’s t-test, Wilcoxon rank-sum test, or χ ^2^ test. A Linear Regression Scoring Method was employed to evaluate the evolution of clinical parameters across the Low muscle spectrum. To identify the core drivers within the interaction network of thyroid hormones, cytokines, and muscle indices, we utilized Network Analysis (GGM). Partial correlation coefficients were calculated to filter confounding factors, and Strength Centrality was used to determine the “bridge” role of IL-6 and TNF-α. To account for individual variability and time effects, a Multivariable Longitudinal Analysis (LMM) was constructed (Random Intercept Model) to assess the impact of fluctuating hormone levels and cytokines on SMI. To explore the relative predictive contributions of changes in thyroid function and inflammatory markers to ΔSMI, an exploratory random forest regression model was fitted in participants with complete paired measurements. Variable changes were defined as the 6-month value minus the baseline value. The model used permutation-based variable importance and was evaluated using repeated cross-validation. Because of the limited sample size, this analysis was considered hypothesis-generating. The Importance Score was calculated to rank the predictive power of ΔIL-6, ΔTNF-α, and ΔThyroid hormones. All analyses were performed using R or Python. *P* < 0.05 was considered statistically significant.

## 3 Results

### 3.1 Baseline data comparison

Of the 369 patients screened, 202 fulfilled the diagnostic criteria for Graves’ hyperthyroidism and 157 completed the baseline assessment and were included in the cross-sectional analysis. Their ages ranged from 18 to 80 years. Among these participants, 80 completed the 6-month follow-up and were included in the longitudinal analysis, the age ranges from 18 to 80 years old, and the median serum thyroid hormone concentrations were FT3 [16.99(10.65, 25.04)] pmol/L, FT4 [41.30(34.22, 61.92)] pmol/L and TSH [0.003(0.002, 0.008)] µU/L. A total of 36 patients with Low muscle mass and 16 patients with sarcopenia were detected. Serum T4 levels in patients with sarcopenia are significantly higher than those in patients with non-sarcopenia (*z* = 2.047; *P* = 0.041), serum IL-6 and Serum TNF-α in sarcopenia and Low muscle mass patients were significantly higher than those in non-sarcopenia patients (*P* < 0.05) (See **Table 1**). FT3, T3 and T4 are negatively correlated with SMI (*r* = -0.16, *r* = -0.20 and r=-0.27; *P* < 0.05). Serum IL-6 and Serum TNF-α were positively correlated with FT3, FT4, T3 and T4 (*P* < 0.05), and negatively correlated with SMI (*r* = -0.19 and *r* = -0.23; *P* < 0.05). There is a positive correlation between Serum IL-6 and Serum TNF-α (*r* = 0.79; *P* < 0.001).

**Table 1:** Main baseline data for all subjects (x̄±*SD or* IQR).

Variable	Non-Sarcopenic	Pre-Sarcopenia	Sarcopenia
Age (years)	47.66±14.42	41.83±15.45	62.50(49.75,66.75)^ab^
Gender	18/87	13/23^a^	4/12
SMI (Kg/m^2^)	6.25(5.84,7.08)	5.20(4.81,6.42)^a^	5.18(4.98,5.37)^a^
ASM(Kg)	16.22(14.68,18.83)	13.59(12.17,17.50)^a^	13.23(11.89,14.88)^a^
Grip(Kg)	29.70±8.71	32.56±7.45	15.00±5.21^ab^
Skeletal muscle mass (Kg)	36.64(35.00,41.65)	32.87(29.46,42.28)^a^	34.22±6.81^a^
BMI (Kg/m^2^)	24.00±3.29	21.11±2.57	22.74±5.26
Serum IL-6(pg/mL)	9.96(6.30,18.06)	15.87(9.90,27.45)^a^	15.81(8.60,28.37)^a^
Serum TNF-α(pg/mL)	15.10(9.34,25.15)	23.04(12.20,40.19)^a^	25.24(15.29,31.85)^a^
FT3 (pmol/L)	16.10(10.41,22.77)	20.24(12.96,30.75)^a^	20.40(7.74,29.31)^a^
FT4 (pmol/L)	39.36(34.34,55.08)	57.16(34.85,85.06)^a^	50.90(27.45,96.16)^a^
TSH (mIU/L)	0.003(0.002,0.008)	0.005(0.003,0.010)	0.005(0.002,0.009)
TG (mmol/L)	1.24±0.73	1.07±0.46	1.31±0.58
TC (mmol/L)	3.88±1.44	3.44±1.31	3.91±1.21
LDL-C (mmol/L)	2.02±0.80	1.92±0.73	2.19±0.61
HDL-C (mmol/L)	1.24±0.32	1.17±0.29	1.32±0.33
GLU (mmol/L)	5.46±3.14	5.43±3.11	5.34±1.52
SBP (mmHg)	126.03±16.01	122.53±10.66	127.38±18.30
DBP (mmHg)	80.88±10.69	80.75±8.72	79.81±11.23

SMI, Skeletal Muscle Index; ASM, Appendicular Skeletal Muscle Mass; BMI, Body Mass Index; IL-6, Interleukin-6; TNF-α, Tumor Necrosis Factor-α; FT3, Free Triiodothyronine; FT4, Free Thyroxine; TSH, Thyroid Stimulating Hormone; TG, Triglycerides; TC, Total Cholesterol; LDL-C, Low-Density Lipoprotein Cholesterol; HDL-C, High-Density Lipoprotein Cholesterol; GLU, Glucose; SBP, Systolic Blood Pressure; DBP, Diastolic Blood Pressure.

^a^ compared with non-sarcoidosis patients in the observation group, ^b^compared with patients with pre-sarcopenia in the observation group, ^C^ compared with the control group.

### 3.2 Gaussian graphical model (GGM) for interaction network analysis

**Figure 2:**
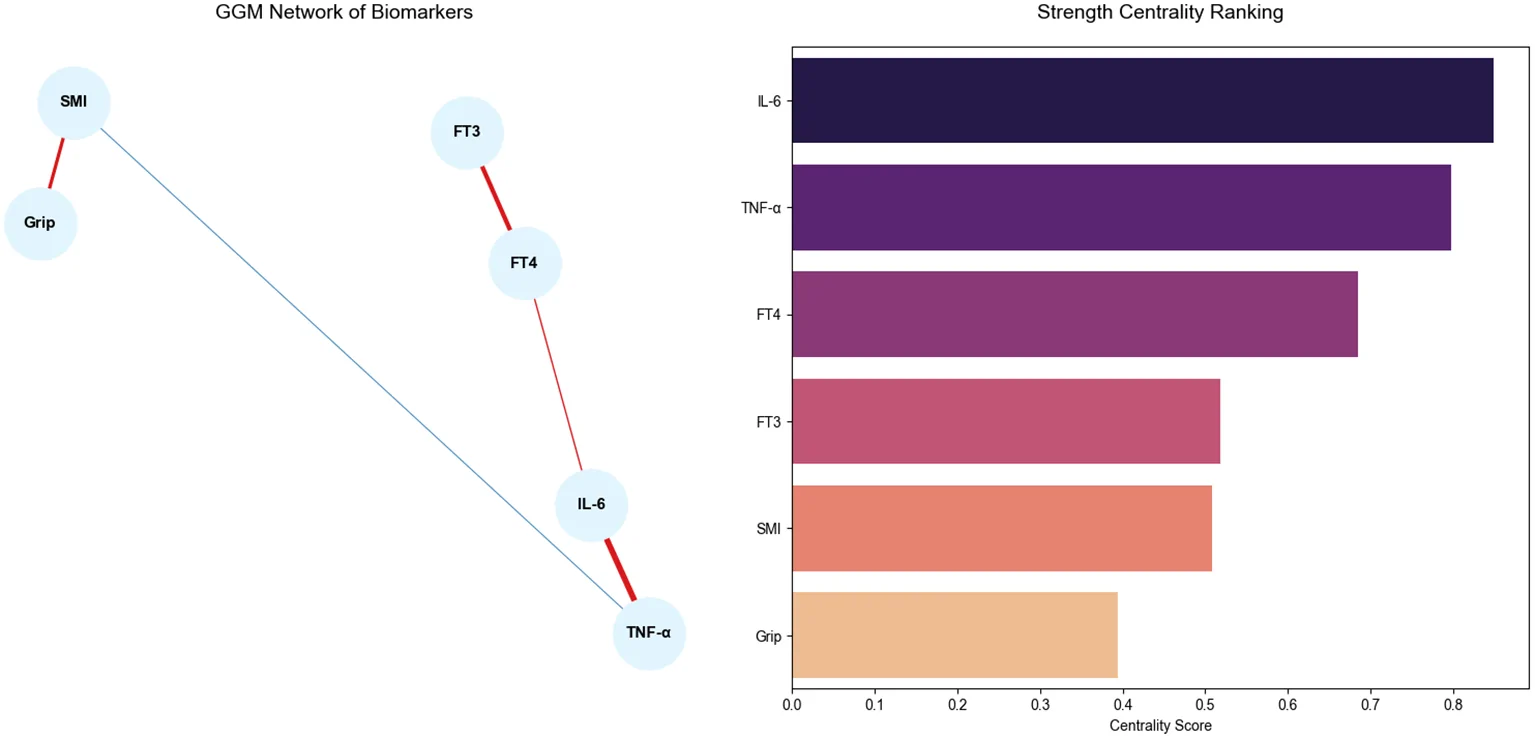
GGM network and strength centrality ranking of biomarkers in patients with thyrotoxic myopathy. (Left) Nodes represent key biomarkers: SMI, Grip, FT3, FT4, IL-6, and TNF-a. Edges represent estimated partial correlations retained by the specified network-estimation procedure; blue and red edges indicate positive and negative associations, respectively. Edge presence should not be interpreted as evidence of causality or statistical significance unless edge-specific uncertainty or significance testing was performed. (Right) Strength centrality ranking of biomarkers. Bars represent strength centrality scores (higher values indicate greater connectivity in the network).

The GGM was employed to identify direct associations between variables by calculating partial correlation coefficients. Strength centrality analysis indicated that IL-6 (centrality = 0.849) and TNF-α (0.797) occupied the most central positions in the interaction network, serving as pivotal “bridges” or “hubs.” Among thyroid hormones, the centrality of FT4 (0.685) was notably higher than that of FT3 (0.518). SMI and handgrip strength showed lower strength-centrality values of 0.508 and 0.394, respectively. This exploratory network indicates that IL-6 and TNF-α were more highly connected to the measured variables, but it does not establish that inflammatory markers causally drive muscle outcomes. (See **Figure 2**).

### 3.3 Exploratory trends across operational muscle-status categories

Trend test analysis using the linear regression scoring method revealed that clinical phenotypes and biochemical parameters evolved significantly across the ordered operational muscle-status categories (from non-sarcopenia to Low muscle mass and sarcopenia groups). Variables included in the trend analyses were prespecified on the basis of clinical relevance and the study hypothesis rather than selected solely from the exploratory GGM. Regarding muscular indices, both skeletal muscle mass index (SMI) (*β*=−0.7190, *P* < 0.001) and handgrip strength (Grip) (*β*=−4.3978, *P* < 0.001) exhibited a significant stepwise decline. Furthermore, BMI decreased significantly with disease progression (*β*=−1.2840, *P* = 0.0023), reflecting the overall metabolic consumption characteristic of sarcopenia under a hyperthyroid background. Systemic inflammation showed a significant positive linear association with sarcopenia severity. Specifically, levels of IL-6 (*β* = 3.5638, *P* = 0.0055) and TNF-α (*β* = 4.9535, *P* = 0.0246) increased significantly as the condition worsened. In terms of thyroid function, FT4 levels showed a significant upward trend (*β* = 11.4277, *P* = 0.0284), whereas TSH and conventional metabolic parameters (including lipid profile, blood pressure, and fasting blood glucose) remained stable across groups without significant linear trends (all *P*>0.05).

### 3.4 Multivariable mixed linear model (LMM) for longitudinal analysis

The IL-6 mixed-effects model included 80 participants and 160 observations. The within-participant association between IL-6 and SMI was not statistically significant (*β* = 0.001, 95%CI=−0.048 to 0.051; *P* = 0.953). The between-participant association of mean IL-6 with SMI was also nonsignificant (*β*=−0.120, 95%CI=−0.274 to 0.033; *P* = 0.128), as was the overall time effect (*β* = 0.585, 95%=CI −0.173 to 1.343; *P* = 0.134). The TNF-α mixed-effects model also included 80 participants and 160 observations. Neither the within-participant association of TNF-α with SMI (*β*=−0.004, 95%CI=−0.050 to 0.041; *P* = 0.849) nor the between-participant association of mean TNF-α with SMI (*β*=−0.038, 95%CI=−0.186 to 0.109; *P* = 0.614) was statistically significant. The time effect was also nonsignificant (*β* = 0.598, 95%CI=−0.131 to 1.327; *P* = 0.112). Neither model showed a singular fit. Continuous inflammatory and thyroid-function variables included in the mixed-effects analyses were standardized; therefore, their coefficients represent the mean difference in SMI associated with a one-standard-deviation difference in the corresponding variable.

### 3.5 Predictive modeling of muscle recovery (random forest and correlation)

Out of the 80 participants who underwent the 6-month longitudinal follow-up, 29 were identified with subnormal muscle levels. Among the 29 participants with low muscle mass at baseline, 7 (24.1%) no longer met the low-muscle-mass criterion at 6 months, whereas 22 (75.9%) continued to meet the criterion. Because all 29 participants had low muscle mass at baseline, there were seven transitions from low to normal muscle mass and no transitions in the opposite direction within this selected subgroup. The exact McNemar test yielded *P* = 0.0156. Both groups showed a significant elevation in SMI compared to pre-treatment values. Additionally, significant disparities were noted between the two groups regarding post-treatment SMI and inflammatory markers, including IL-6 and TNF-α (**Table 2**).

**Table 2:** Comparison of baseline characteristics between low muscle mass group and normal group participants after treatment (x̄±*SD or* IQR).

Variable	Low muscle mass	Normal	*t*/*z*/*x^2^*	*P*
Gender	11/18	12/39	1.872	0.171
SMI (Kg/m^2^)Gender	5.35(5.01,6.68)	6.46(6.06,7.63)	3.678	<0.001
Skeletal muscle mass (Kg)	33.91(30.79,44.30)	37.90(35.36,47.67)	2.797	0.005
Serum IL-6(pg/mL)	20.45(13.06,25.60)	8.89(4.39,15.46)	3.958	<0.001
Serum TNF-α(pg/mL)	21.41(13.96,32.98)	15.70(7.20,27.30)	1.997	0.048
FT3 (pmol/L)	3.93±1.12	3.67±1.25	0.934	0.353
FT4 (pmol/L)	13.11±4.22	14.58±4.26	1.489	0.140
TSH (mIU/L)	2.13(1.42,2.38)	2.04(1.32,2.90)	0.420	0.674

SMI, Skeletal Muscle Index; IL-6, Interleukin-6; TNF-α, Tumor Necrosis Factor-α; FT3, Free Triiodothyronine; FT4, Free Thyroxine; TSH, Thyroid Stimulating Hormone.

**Figure 3:**
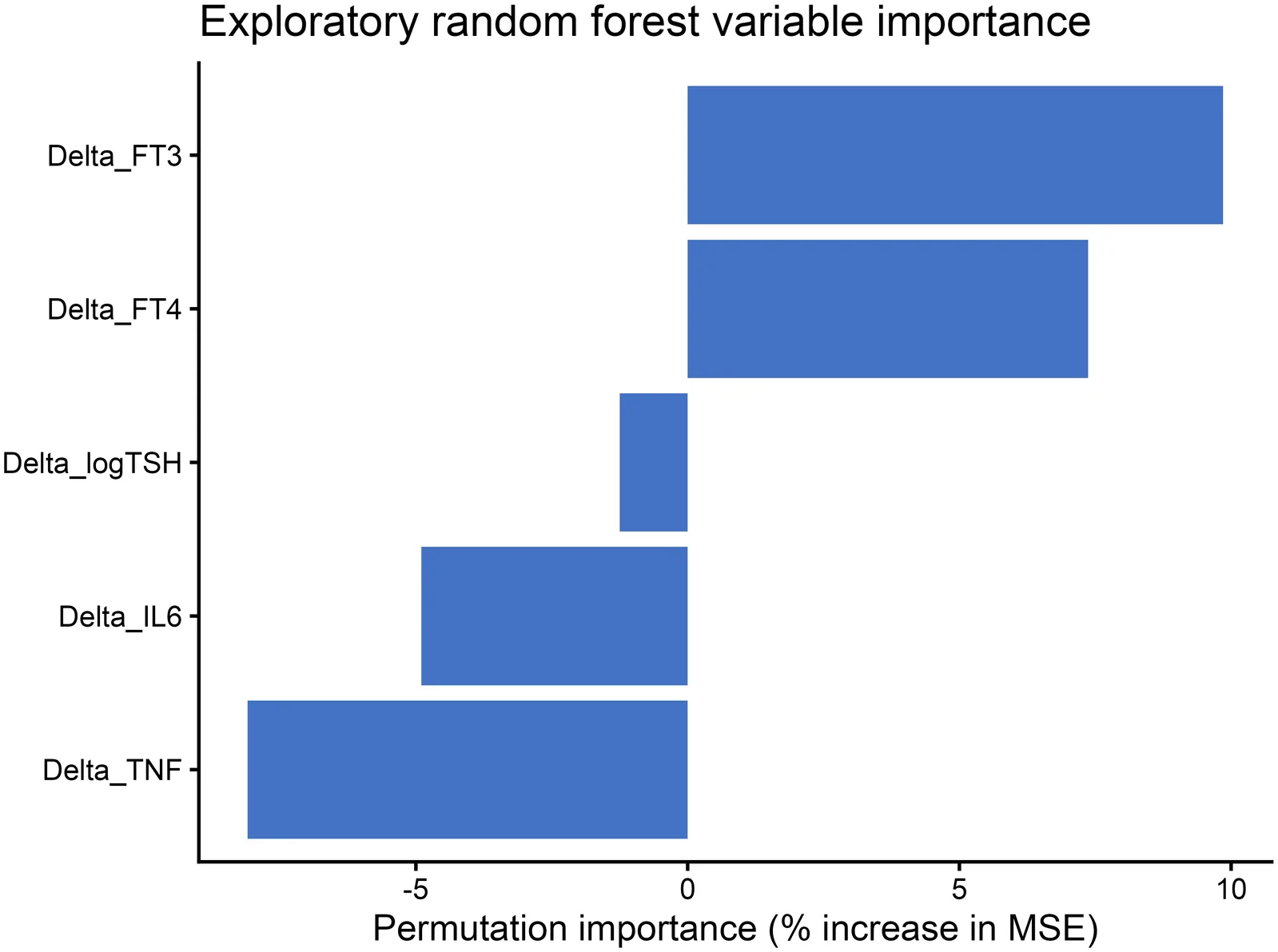
Feature importance rankings in Random Forest models for ΔSMI prediction. The random forest model included 80 complete cases. Change variables were calculated as the 6-month post-treatment value minus the baseline value. Permutation importance was expressed as the percentage increase in mean squared error after permutation of each variable. The mean performance across 20 repetitions of five-fold cross-validation was RMSE = 0.450 (SD 0.013), MAE = 0.344 (SD 0.012), and R²=−0.204 (SD 0.072), indicating limited out-of-sample predictive performance. Variable importance represents an exploratory ranking and should not be interpreted as an independent or causal effect. Negative importance values indicate that permuting the corresponding variable did not reduce predictive performance.

The exploratory random forest analysis included 80 complete cases. Across 20 repetitions of five-fold cross-validation, the mean RMSE was 0.450 (SD 0.013), the mean MAE was 0.344 (SD 0.012), and the mean R² was −0.204 (SD 0.072). The negative cross-validated R² indicates that the model had limited out-of-sample predictive performance and did not outperform a simple mean-based prediction in the validation folds. In the final model fitted to the complete dataset, the three highest permutation-importance values were observed for ΔFT3 (9.85), ΔFT4 (7.37), and Δlog-TSH (−1.25). These values represent exploratory variable rankings only. In particular, a negative permutation-importance value indicates that permuting the variable did not worsen—and may have slightly improved—model prediction. Therefore, the variable-importance ranking should not be interpreted as evidence of an independent association, causal effect, or clinically useful predictive contribution. (See **Figure 3**).

**Figure 4:**
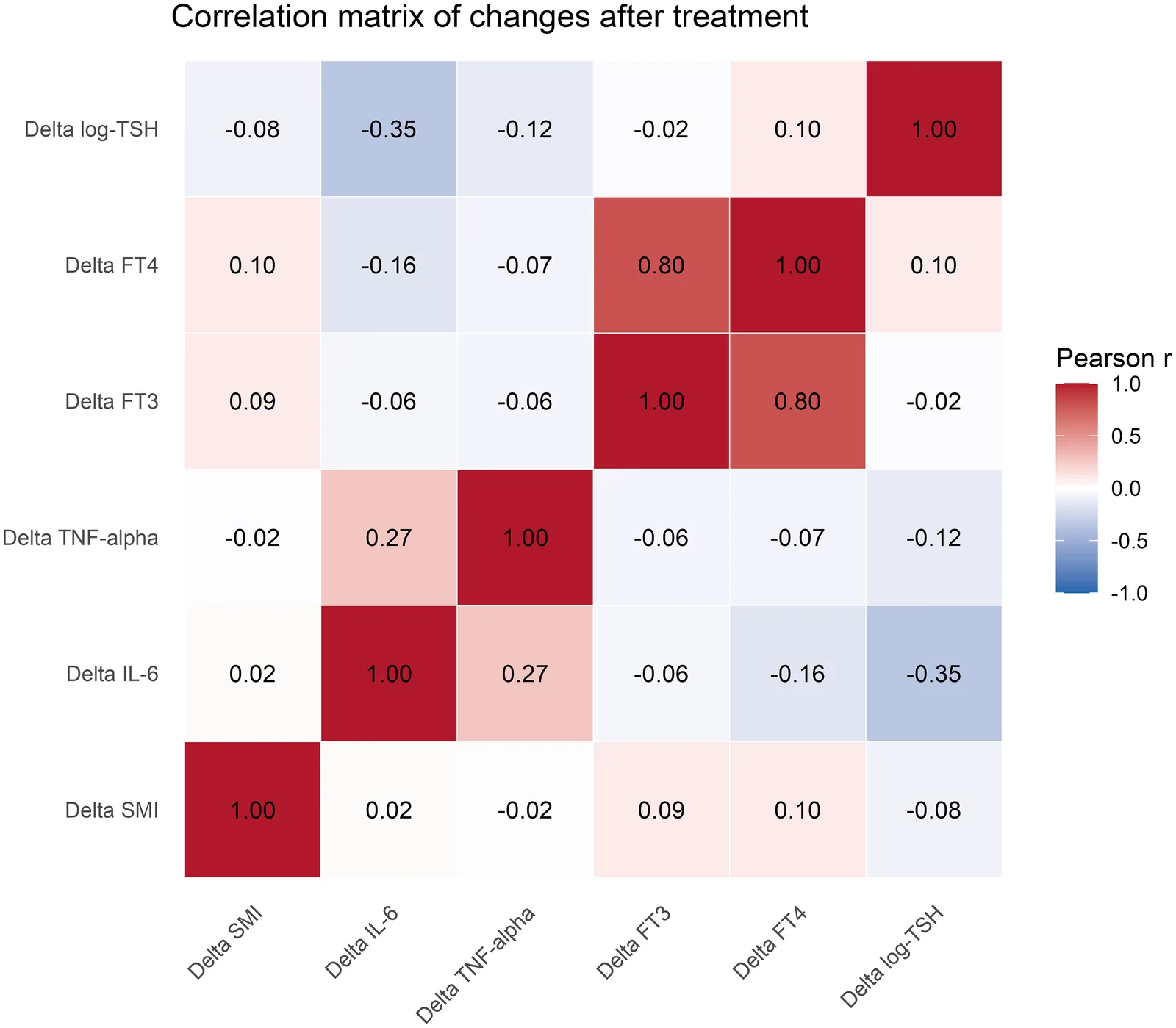
Pearson correlation matrix of changes in SMI, inflammatory markers, and thyroid-function measures during follow-up. All change variables were calculated as the 6-month post-treatment value minus the baseline value. The displayed values are Pearson correlation coefficients. None of the correlations between ΔSMI and ΔIL-6, ΔTNF-α, ΔFT3, ΔFT4, ΔTSH, or Δlog-TSH was statistically significant after Benjamini–Hochberg correction.

Pearson and Spearman correlation analyses did not identify statistically significant associations between changes in SMI and changes in IL-6, TNF-α, FT3, FT4, TSH, or log-transformed TSH after Benjamini–Hochberg correction. Specifically, ΔIL-6 was not correlated with ΔSMI (Pearson r=0.020, 95%CI=−0.201 to 0.238, P = 0.863, BH-adjusted P = 0.877; Spearman ρ=−0.013, P = 0.908, BH-adjusted P = 0.908; n=80). Similarly, ΔTNF-α was not correlated with ΔSMI (Pearson r=−0.018, 95%CI=−0.236 to 0.203, P = 0.877, BH-adjusted P = 0.877; Spearman ρ=0.104, P = 0.360, BH-adjusted P = 0.583; n=80). The Pearson correlation coefficients for ΔFT3 and ΔFT4 with ΔSMI were 0.095 (95%CI=−0.128 to 0.308; BH-adjusted P = 0.751) and 0.098 (95%CI=−0.125 to 0.311; BH-adjusted P = 0.751), respectively. Although the Spearman correlation between ΔTSH and ΔSMI was nominally significant before correction (ρ=−0.243, P = 0.030), it was no longer significant after BH correction (BH-adjusted P = 0.180). The corresponding Pearson association for ΔTSH and both the Pearson and Spearman associations for Δlog-TSH were also nonsignificant. In prespecified multivariable linear regression analyses, neither ΔIL-6 nor ΔTNF-α was independently associated with ΔSMI. For ΔIL-6, the adjusted regression coefficient was 0.009 (95%CI=−0.018 to 0.036; P = 0.512; BH-adjusted P = 0.825; n=80). For ΔTNF-α, the adjusted regression coefficient was 0.001 (95%CI=−0.011 to 0.013; P = 0.825; BH-adjusted P = 0.825; n=80). Each model adjusted for the baseline value of the corresponding inflammatory marker, ΔFT4, Δlog-TSH, baseline SMI, age, sex, and baseline BMI. These estimates do not support an independent association between changes in either inflammatory marker and changes in SMI in the present sample. (See **Figure 4**).

### 3.6 Exploratory linear and nonlinear associations of changes in inflammatory markers with ΔSMI

**Figure 5:**
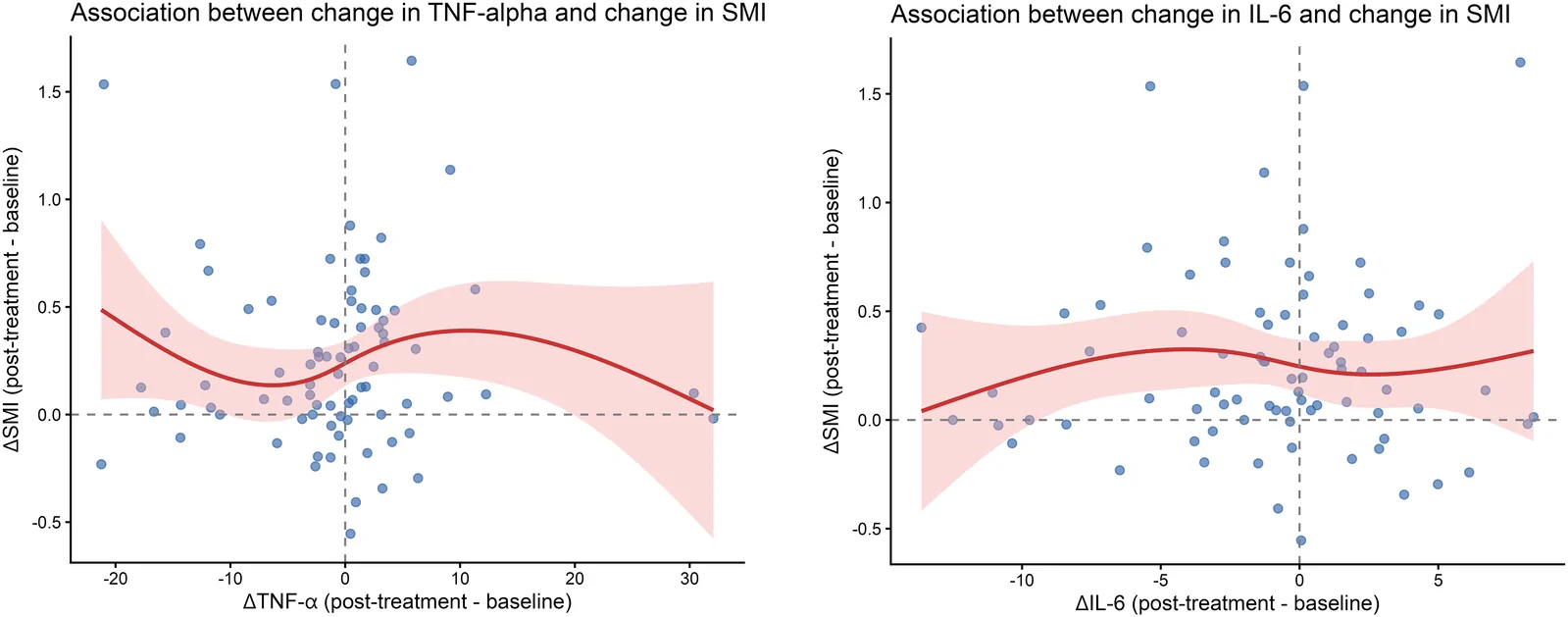
Exploratory associations of changes in TNF-α and IL-6 with changes in skeletal muscle mass index. **(A)** Association between ΔTNF-α and ΔSMI. **(B)** Association between ΔIL-6 and ΔSMI.

The associations of ΔIL-6 and ΔTNF-α with ΔSMI were evaluated using adjusted linear models and natural spline models. The nonlinear analyses were exploratory, and the spline models were compared with the corresponding linear models to determine whether allowing nonlinearity significantly improved model fit. (See **Figure 5**).

Scatter plots for both ΔTNF-α (**Figure 5A**) and ΔIL-6 (**Figure 5B**) revealed that the majority of observations were highly concentrated around the origin (0,0). This cluster pattern suggests that within the typical range of clinical fluctuation during treatment, minor changes in inflammatory markers do not translate into a strong, direct linear change in muscle mass. For ΔIL-6, the linear association with ΔSMI was not statistically significant (*P* = 0.512). The natural spline model did not provide a significantly better fit than the linear model (likelihood-ratio/model-comparison *P* = 0.576; BH-adjusted *P* = 0.576). The AIC was 101.0 for the linear model and 103.7 for the spline model. For ΔTNF-α, the linear association with ΔSMI was also nonsignificant (*P* = 0.825), and the natural spline model did not significantly improve model fit compared with the linear model (model-comparison *P* = 0.402; BH-adjusted *P* = 0.576). The corresponding AIC values were 101.5 and 103.3, respectively. Thus, neither analysis provided sufficient evidence of a nonlinear or threshold association between changes in inflammatory markers and changes in SMI. The wider confidence intervals at the extremes primarily indicate sparse data and increased estimation uncertainty and should not be interpreted as evidence of a threshold effect.

## 4 Discussion

The present prospective longitudinal study investigates the “thyroid-inflammation-muscle” axis in patients with Graves’ hyperthyroidism undergoing radioactive iodine (RAI) therapy. Our findings showed that IL-6 and TNF-α were associated with adverse muscle-status phenotypes at baseline, whereas their longitudinal changes were not significantly associated with changes in SMI in the adjusted mixed-effects model. The exploratory random forest analysis ranked ΔFT3 and ΔFT4 as the two most important variables, whereas Δlog-TSH had negative permutation importance and changes in inflammatory markers did not show robust predictive contributions. Moreover, the negative cross-validated R² indicated poor out-of-sample predictive performance; therefore, these rankings should be considered hypothesis-generating only. These findings indicate that inflammatory markers are associated with muscle status cross-sectionally, but they do not demonstrate that changes in inflammation independently determine muscle recovery after treatment. Traditionally, muscle wasting in chronic hyperthyroidism has been attributed to accelerated protein catabolism. Excessive thyroid hormones promote the release of amino acids from skeletal muscle, thereby stimulating protein decomposition and leading to a reduction in muscle mass (4). Beyond these metabolic shifts, thyroid hormones exert direct effects on muscle fiber integrity and contractile function. High levels of thyroxine (T4) have been shown to impair muscle contractility, while triiodothyronine (T3) serves as a critical regulator of myosin heavy chain (MHC) isoforms. Specifically, T3 stimulates the expression of Myh1, Myh2, and Myh4 while inhibiting Myh7 (12, 13). Consequently, excessive or dysregulated T3 signaling may alter MHC isoform expression and muscle-fiber composition, potentially compromising skeletal muscle contractile function. Furthermore, T3 influences muscle regeneration by modulating the proliferation and differentiation of progenitor cells; however, pathologically high T3 levels can paradoxically inhibit satellite cell proliferation and promote premature differentiation, thereby hindering muscle repair (14). IL-6 and TNF-α are among the most extensively studied pro-inflammatory mediators associated with muscle loss in several clinical settings. Nevertheless, skeletal muscle wasting is regulated by a broader network of cytokines, chemokines, myokines, and metabolic mediators, and the present study did not determine whether IL-6 and TNF-α have a predominant role within this network. While previous research by Sheng et al. (32) and Bertoli et al. (33) observed that lower FT3 levels correlate with muscle weakness in the context of non-thyroidal illness syndrome (NTIS), the muscle wasting observed in our hyperthyroid cohort is distinct, primarily driven by the thyrotoxicosis-induced inflammatory surge. Reductions in inflammatory markers and improvement in SMI were observed during the same follow-up period; however, temporal co-occurrence does not establish an association, mediation, or causality. Changes in IL-6 and TNF-α were not significantly associated with ΔSMI in the correlation analyses, adjusted linear regression models, or mixed-effects models. Therefore, the present findings do not support the use of IL-6 or TNF-α as validated longitudinal biomarkers of muscle recovery.

Data from the GGM demonstrated high strength centrality for IL-6 and TNF-α. In the interaction network, these cytokines showed relatively high conditional connectivity with the measured thyroid and muscle-related variables. Because the network was cross-sectional, it cannot determine temporal ordering, mediation, or causality. While this mechanism is well-documented in the “inflammaging” paradigm, where chronic elevation of pro-inflammatory cytokines such as TNF-α and IL-6—primarily secreted by M1 macrophages—is directly linked to the degradation of muscle mass and strength (34), our data extends this observation to thyrotoxicosis in non-geriatric populations. We hypothesize that elevated FT4 levels initiate a systemic inflammatory cascade; TSH itself upregulates IL-6 through cyclic adenosine monophosphate (cAMP) signaling in thyroid and non-thyroid cells (35). Animal studies link T3-induced oxidative stress to enhanced nuclear factor-κB (NF-κB) activity and subsequent TNF-α and IL-10 expression (36, 37). Chronic low-grade inflammation driven by IL-6 and TNF-α promotes muscle proteolysis, inhibits protein synthesis, and induces insulin resistance—mechanisms implicated in sarcopenia (18, 25). IL-6 impairs insulin-dependent glycogen synthesis in liver and adipocytes while paradoxically enhancing it in myotubes; TNF-α stimulates lipolysis, elevates free fatty acids, and reduces muscle glucose uptake, collectively fostering insulin resistance commonly observed in hyperthyroidism (38). Systemic inflammation and insulin resistance exacerbate catabolism, suppress anabolism, and ultimately drive muscle wasting (39, 40). Subsequently, IL-6 and TNF-α may facilitate myofibrillar protein degradation, potentially through the activation of the ubiquitin-proteasome pathway (UPP) or inhibition of IGF-1 signaling. The critical role of thyroid hormones in modulating the IGF-1/PI3K/Akt signaling axis is supported by recent evidence showing that thyroid status directly regulates IGF-1 expression and its downstream protective pathways in striated muscles (41). SMI and handgrip strength had lower strength-centrality estimates than the inflammatory markers. This reflects their lower connectivity in the fitted network but does not establish that muscle abnormalities are secondary consequences of cytokine changes.

Changes in IL-6 and TNF-α were not significantly associated with ΔSMI in the correlation, adjusted linear regression, or mixed-effects analyses. Biological systems often exhibit non-linear characteristics that traditional linear models may fail to capture (42). Random forest models can capture complex interactions and nonlinear patterns, but such flexibility may also increase overfitting in small datasets and requires assessment using out-of-sample validation (43). The clustering of observations near the origin indicates that most participants experienced relatively limited simultaneous changes in inflammatory markers and SMI. Sparse observations at the extremes resulted in substantial uncertainty and did not provide evidence of a threshold association. One possible explanation for the discrepancy between random forest importance and weak linear associations is the presence of interactions or nonlinear patterns. However, the formal spline analyses did not provide statistically significant evidence of nonlinearity, and the sparse data at the extremes precluded reliable estimation of a threshold. The random forest findings should therefore be interpreted as exploratory. Cytokines such as IL-6 and TNF-α exhibit a classic dose-dependent or biphasic response in skeletal muscle: physiological/low levels promote satellite cell activation and muscle regeneration, whereas chronically elevated levels drive proteolysis and muscle wasting—a phenomenon often described as a “double-edged sword” (44, 45). In the present dataset, the random forest model did not demonstrate reliable out-of-sample predictive performance, and therefore it cannot be considered evidence that such interactions were identified. ΔFT3 and ΔFT4 had the highest permutation-importance values in the exploratory random forest model. However, because the model had a negative cross-validated R² and the corresponding correlation analyses were nonsignificant, these rankings do not establish predictive utility, statistical significance, or causality. Although FT3 and FT4 often normalize shortly after RAI (46), the delay in TSH recovery, frequently persisting for more months despite euthyroid or hypothyroid peripheral hormone levels, is a well-recognized phenomenon reflecting prolonged suppression of pituitary thyrotrophs and the time required for deeper metabolic recalibration beyond mere hormone normalization (47). This extended phase allows for concurrent suppression of hypermetabolism and resolution of systemic inflammation, as evidenced by cumulative progression toward stable thyroid function over time. Our results suggest that SMI restoration is not solely a consequence of reduced thyroid hormone levels but involves a broader homeostatic recovery, where the suppression of hypermetabolism and the resolution of inflammation occur concurrently (48). From a clinical perspective, direct assessment of muscle mass and function may provide information that is not fully captured by thyroid-function tests alone. However, the present findings do not demonstrate that measuring IL-6 or TNF-α improves the prediction of muscle recovery beyond routine thyroid-function tests and direct muscle assessment. The incremental clinical value of these or other inflammatory mediators should be evaluated in larger prospective cohorts using predefined multivariable models, broader biomarker panels, and external validation.

Several limitations should be acknowledged. First, only 80 of the 157 baseline participants had complete paired data at 6 months, creating a risk of attrition bias despite comparison of baseline characteristics between completers and non-completers. Second, the subgroup with low muscle mass at baseline included only 29 participants, limiting statistical power and the stability of machine-learning and nonlinear analyses. Third, gait speed was not measured, and the AWGS thresholds were applied to a cohort spanning 18–80 years; therefore, the muscle-status categories should not be interpreted as definitive diagnoses of primary age-related sarcopenia. Fourth, the follow-up period included only two measurement occasions and cannot characterize the temporal sequence of thyroid normalization, cytokine changes, and muscle recovery. Fifth, residual confounding from dietary intake, protein consumption, physical activity, resistance exercise, sex hormones, vitamin D, IGF-1, antithyroid medication, levothyroxine replacement, and post-RAI thyroid status cannot be excluded. Sixth, the random forest and spline analyses were exploratory and lacked external validation. Finally, the observational design does not permit causal or mediation inferences regarding inflammatory markers and skeletal muscle recovery.

## 5 Conclusion

In conclusion, some patients with Graves’ hyperthyroidism showed recovery of skeletal muscle mass during the 6 months after RAI treatment; among the 29 participants with low muscle mass at baseline, 7 no longer met the low-muscle-mass criterion at follow-up. IL-6 and TNF-α were associated with adverse muscle-status phenotypes at baseline, but their longitudinal associations with SMI recovery were not statistically significant in the adjusted mixed-effects analysis. The exploratory random forest ranked ΔFT3 and ΔFT4 highest, but its negative cross-validated R² indicated no reliable out-of-sample predictive performance. Therefore, no evaluated thyroid or inflammatory change marker can currently be considered a clinically useful predictor of SMI recovery. The present study does not establish a causal or threshold effect of IL-6 or TNF-α on muscle recovery.

## Data Availability

The raw data supporting the conclusions of this article will be made available by the authors, without undue reservation.
